# Faricimab: current evidence for the treatment of retinal vein occlusion

**DOI:** 10.1007/s00417-026-07304-2

**Published:** 2026-06-10

**Authors:** Livio Vitiello, Giuseppe Giannaccare, Enrico Borrelli, Giulia Coco, Flavia Chiosi, Filippo Lixi, Mario Troisi, Vincenzo Gagliardi, Eleonora D’Aniello, Alfonso Pellegrino, Claudio Iovino

**Affiliations:** 1 Eye Unit, “Luigi Curto” Hospital, Azienda Sanitaria Locale Salerno, Polla, 84035 Italy; 2 Eye Clinic, Department of Surgical Sciences, University of Cagliari, Cagliari, 09124 Italy; 3 Department of Surgical Sciences, University of Turin, Turin, 10124 Italy; 4 Department of Ophthalmology, City of Health and Science Hospital, Turin, 10126 Italy; 5 Ophthalmology Unit, Department of Clinical Sciences and Translational Medicine, University of Rome Tor Vergata, Rome, 00133 Italy; 6 Department of Ophthalmology, Azienda Ospedaliera dei Colli, Monaldi Hospital, Napoli, 80131 Italy; 7 Ophthalmologic Unit, University Hospital of Salerno, Salerno, 84100 Italy; 8 Villa dei Fiori, Acerra, 80011 Italy

**Keywords:** Faricimab, BRVO, CRVO, HRVO, Retinal vein occlusion, RVO.

## Abstract

**Purpose:**

To provide an updated and critical overview of the available clinical evidence on the use of faricimab for the treatment of macular edema secondary to retinal vein occlusion (RVO), with particular focus on efficacy, durability of response, and clinical applicability in both trial and real-world settings.

**Methods:**

A systematic literature review was performed in accordance with the Preferred Reporting Items for Systematic Reviews and Meta-Analyses (PRISMA) guidelines using PubMed and Scopus databases. Pivotal phase III randomized clinical trials and real-world studies evaluating intravitreal faricimab in patients with branch, central, or hemiretinal vein occlusion were included. Both treatment-naïve and previously treated eyes were considered. Evidence was qualitatively synthesized with attention to visual and anatomical outcomes, treatment durability, and safety.

**Results:**

Data from the BALATON and COMINO phase III trials demonstrated that faricimab achieved visual and anatomical outcomes comparable to aflibercept 2.0 mg during fixed monthly dosing, with maintenance of efficacy up to 72 weeks under treat-and-extend regimens. A clinically meaningful proportion of patients achieved extended treatment intervals, suggesting potential reduction in treatment burden. Real-world studies largely confirmed consistent anatomical efficacy across different RVO subtypes and clinical scenarios. However, functional improvement was more heterogeneous, particularly in chronic or therapy-resistant cases, where anatomical and visual outcomes were frequently dissociated, likely reflecting irreversible retinal ischemia or structural damage. Across studies, faricimab showed a generally favorable safety profile.

**Conclusion:**

Available evidence supports faricimab as an effective and well-tolerated therapeutic option for RVO-related macular edema, especially when initiated early in the disease course. However, although extended dosing intervals were achieved in a proportion of patients, superiority in durability compared with other anti-VEGF agents has not yet been demonstrated. Further prospective, comparative, and long-term real-world studies are needed to better define patient selection criteria and the optimal positioning of faricimab within current RVO treatment algorithms.

## Key Messages

***What is known*:**

- Anti-vascular endothelial growth factor (VEGF) agents are the standard first-line treatment for macular edema secondary to retinal vein occlusion (RVO), and faricimab has recently expanded available therapeutic options through dual VEGF-A and angiopoietin-2 inhibition.

- Evidence from phase III trials showed that faricimab achieves visual and anatomical outcomes comparable to aflibercept 2.0 mg, with sustained efficacy under treat-and-extend regimens and the possibility of extending treatment intervals.

***What is new*:**

- Real-world studies confirmed good anatomical responses to faricimab in both treatment-naïve and previously treated RVO patients, although visual outcomes were more heterogeneous, particularly in chronic or therapy-resistant disease.
- The dissociation between anatomical and functional outcomes observed in advanced RVO highlights the importance of early treatment initiation and careful patient selection to optimize clinical benefit.

## Introduction

Retinal vein occlusion (RVO) is currently the second most frequent cause of vision loss associated with retinal vascular disease [1]. Despite the difficulty in clinical classification, RVO has historically been divided into two main categories: (I) branch RVO (BRVO), which is characterized by an obstruction within one of the branches of the retinal vein, typically at the site where the retinal artery and vein cross, and (II) central RVO (CRVO), where the obstruction occurs within the central retinal vein located within the optic nerve, thus affecting the entire retina [2].

Due to the incomplete characterization of various elements of the underlying etiology [3], there is continuous debate about the best ways to manage RVO and its complications, including conservative (particularly wait-and-see strategy, since CRVO may also improve spontaneously) [4, 5], medical, and surgical methods. Additionally, new pharmacological options and imaging technologies continue to broaden the diagnostic and therapeutic approaches for various ocular vascular diseases [6–9].

The most common vision-threatening complication of RVO is macular edema, which affects up to 15% of eyes with BRVO and 30% of eyes with CRVO [10, 11]. Nowadays, intravitreal anti-vascular endothelial growth factor (VEGF) therapy is considered the mainstay of treatment for macular edema related to RVO [12]. In fact, persistent vascular blockage can lead to varying degrees of retinal ischemia and macular edema. For this reason, the vascular wall may be harmed by pressure buildup inside the blocked retinal veins, leading to vascular leakage and the development of macular edema. Thus, the sudden, transient retinal ischemia causes the release of VEGF and other inflammatory mediators, which leads to the development of macular edema [13].

Macular edema associated with RVO is often treated with three anti-VEGF drugs: bevacizumab, ranibizumab, and aflibercept [14, 15]. Moreover, intravitreal corticosteroids such as dexamethasone or fluocinolone acetonide implants are also typically utilized as a second-line treatment for this retinal disease [16–18].

However, in the intricate pathophysiology of RVO, VEGF is not the only harmful agent. In fact, in recent years, angiopoietin-2 (Ang-2), which controls vascular stability, inflammation, and permeability, has been also demonstrated to have a critical role [19]. Increased Ang-2 levels in RVO may worsen inflammation and vascular leakage, accelerating the course of the disease.

A new bispecific monoclonal antibody called faricimab (Vabysmo^®^, Genentech/Roche, Switzerland) can target both vascular endothelial growth factor-A (VEGF-A) and Ang-2 at the same time inside a same molecular framework [20, 21]. Therefore, faricimab seeks to provide more powerful and long-lasting anti-permeability and anti-inflammatory effects by co-inhibiting these two crucial signaling pathways. In the treatment of diabetic macular edema and neovascular age-related macular degeneration, faricimab has already demonstrated encouraging efficacy and the ability to extend dosing intervals in both clinical trials and real-world studies [22–24]. More recently, the BALATON and COMINO trials demonstrated the efficacy of faricimab in patients with macular edema secondary to RVO, leading to its approval for this indication [25].

VEGF-A promotes endothelial cell migration, proliferation, and neovascularization in RVO by binding to VEGF receptor-2 (VEGFR-2). VEGF-A overexpression causes aberrant neovascularization and retinal fluid buildup, which causes macular edema and visual loss. Faricimab can inhibit VEGF-A-mediated signaling pathways by binding to VEGF-A and preventing it from interacting with VEGFR-2. This seems to significantly slow the evolution of the disease and enhance visual outcomes by reducing endothelial cell migration, proliferation, and tube formation [26, 27]. In addition, faricimab not only inhibits VEGF-A but also Ang-2, which aggravates retinal damage by disrupting vascular integrity and enhancing the pro-angiogenic effects of VEGF in pathological settings [28]. In this way, faricimab can attenuate VEGF-driven angiogenesis, lower inflammation, and improve vascular barrier function by concurrently blocking the VEGF-A and Ang-2 pathways [20, 29]. For this reason, the bispecific targeting mechanism of faricimab may be of potential relevance in the management of RVO, given the involvement of both VEGF-A and Ang-2 pathways, especially in terms of reducing inflammatory processes, preserving vascular stability, and trying to enhance the retinal microenvironment, which prolongs vascular stability and lowers the chance of disease recurrence [27, 30].

The purpose of this up-to-date review is to provide a comprehensive and methodical overview of the currently available evidence on the use of faricimab in the treatment of RVO, spanning from pivotal registration trials to the most recent real-world data, including both treatment-naïve patients and cases switched from other therapeutic strategies.

## Materials and methods

### Search strategy and study design

This study was conducted as a systematic review of the literature in accordance with the Preferred Reporting Items for Systematic Reviews and Meta-Analyses (PRISMA 2020) guidelines [31]. A comprehensive search of the PubMed (MEDLINE) and Scopus databases was performed to identify studies evaluating the efficacy and safety of faricimab for the treatment of macular edema secondary to RVO.

The literature search was conducted in March 2026, and included all articles published from January 2019 to March 2026 (corresponding to the period during which faricimab became available in the clinical practice).

The following search strategy was used: (“faricimab”) AND (“retinal vein occlusion” OR “RVO” OR “branch retinal vein occlusion” OR “BRVO” OR “central retinal vein occlusion” OR “CRVO” OR “hemiretinal vein occlusion” OR “HRVO”). In addition, the reference lists of the included articles were manually screened to identify additional relevant studies that were not captured in the initial database search.

### Eligibility criteria

Eligible studies included English-language randomized clinical trials and real-world studies (prospective and retrospective) or case series/reports evaluating intravitreal faricimab for macular edema secondary to RVO, reporting functional and/or anatomical outcomes, such as best corrected visual acuity (BCVA) and central subfoveal thickness (CST).

Preclinical or animal studies, editorials, commentaries and studies not providing extractable faricimab-specific data were excluded. Unrelated and duplicated publications and studies evaluating faricimab in retinal diseases other than RVO were also excluded.

### Study selection

All identified records were screened independently by two reviewers (L.V and C.I.) based on title and abstract. Full-text articles were subsequently assessed for eligibility according to the predefined inclusion and exclusion criteria. Any discrepancies were resolved through discussion among the authors until consensus was reached.

**Fig. 1:**
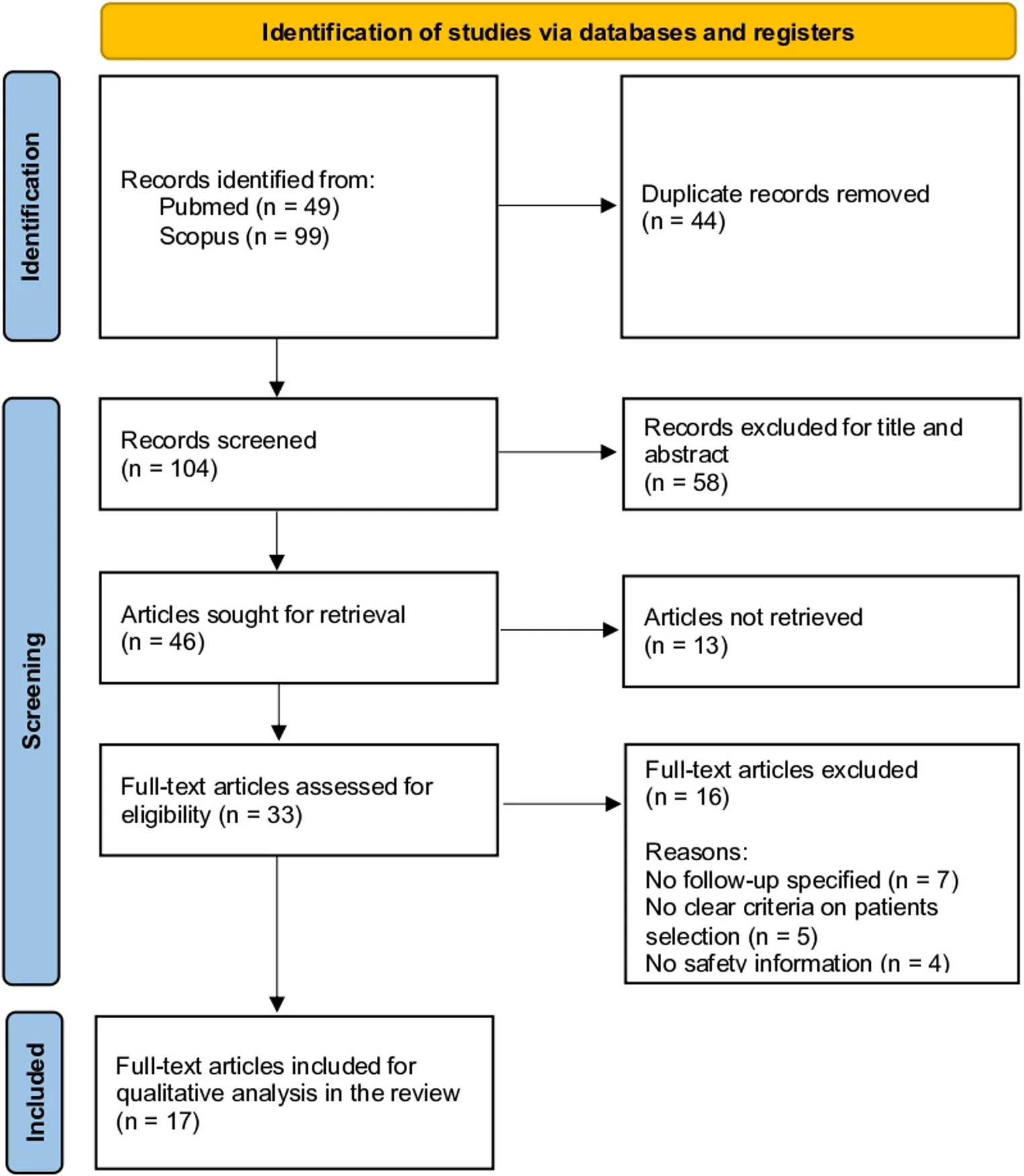
PRISMA flow diagram of study selection

The study selection process is summarized in Fig. 1 using a PRISMA flow diagram.

### Data extraction

For each included study, the following data were extracted: study design, sample size, type of RVO (BRVO, CRVO, HRVO), treatment setting (treatment-naïve or switch therapy), follow-up duration, change in BCVA, change in CST, number of injections, safety outcomes.

Given the heterogeneity in study design, treatment regimens, follow-up duration, and outcome reporting, a formal quantitative meta-analysis was not considered methodologically appropriate. Therefore, the results were qualitatively synthesized and critically analyzed.

### Quality assessment and data synthesis

Due to the limited number of available studies and the marked heterogeneity in methodology, follow-up duration, and outcome measures, a formal risk-of-bias scoring system was not applied. However, each study was critically evaluated based on study design, sample size, duration of follow-up, and clinical relevance of the reported outcomes.

The results were qualitatively synthesized, with particular attention to differences between randomized clinical trials and real-world studies, as well as between treatment-naïve and previously treated patients. At last, 17 articles on the use of faricimab in RVO patients were critically analyzed in this review.

### Registration clinical trials of faricimab for RVO: BALATON and COMINO

Two international multicenter Phase III clinical trials, BALATON and COMINO, are designed to thoroughly validate the safety and efficacy of faricimab in the treatment of RVO [32]. While COMINO focused on CRVO-related macular edema, BALATON assessed the safety and effectiveness of faricimab in treating BRVO-related macular edema [33].

A total of 1282 patients were enrolled in these two trials: 729 in the COMINO study and 553 in the BALATON trial. Participants were randomized to receive injections of either aflibercept 2.0 mg or faricimab 6.0 mg every four weeks for a total of six treatments. According to the trial’s findings, patients in the faricimab group showed significant improvements in their vision after taking the drug every four weeks for up to 24 weeks [33]. In addition, faricimab was also linked to a greater proportion of patients experiencing no retinal leakage. This could imply that faricimab’s dual mechanism of action, which inhibits both VEGF-A and angiopoietin-2, may contribute to the observed anatomical outcomes beyond VEGF inhibition alone, although direct comparative advantages remain to be fully established. This dual mechanism may also enhance anatomical results in RVO patients and treat more properly several aspects of this retinal vascular disease [33].

Subsequently, the study by Danzig et al. [34] reported the 72-week results of the BALATON and COMINO phase III trials, evaluating the efficacy, duration of response, and safety of faricimab in a treat-and-extend (T&E) regimen in RVO patients. The main contribution of this study lies in demonstrating the functional and anatomical sustainability of the response beyond one year. In terms of efficacy, the visual gains and reduction in CST achieved in the initial monthly dosing phase remained stable up to 72 weeks, even after transitioning to a T&E regimen [34]. In this sense, BALATON and COMINO provide randomized phase III evidence supporting the maintenance of visual and anatomical benefits beyond the first year in naïve patients with RVO. Another element of interest is the durability of response, with a clinically significant proportion of patients achieving treatment intervals longer than 12 weeks (over 60% in BRVO and approximately 45–50% in CRVO at week 68) [34]. Although these percentages are lower than those observed in faricimab trials for diabetic macular edema and neovascular age-related macular degeneration [35, 36], this is likely attributable to both the greater severity and biological instability of RVO, particularly CRVO, and the adoption of a more restrictive T&E protocol, which limits re-extensions after anatomical or functional deterioration.

In conclusion, BALATON and COMINO provide substantial evidence supporting the use of faricimab in T&E therapy for RVO, showing that functional and anatomical benefits are maintained beyond one year with a potential decrease in the therapeutic burden. However, interpretation of the results must consider the non-comparative design of the extended phase and the need for further longer-term data in real-world settings to more accurately define the role of faricimab compared to traditional anti-VEGF agents in different forms of RVO.

## Real-world experience with faricimab in RVO patients

After the two global multicenter clinical trials BALATON and COMINO, several research studies were carried out on the use of faricimab in RVO patients to assess its efficacy and safety in a real-life setting, both in naïve patients and in case of switching therapy.

Hikichi and colleagues performed a retrospective observational study comparing the 6-month results of aflibercept 2.0 mg and faricimab in naïve patients with macular edema secondary to BRVO [37]. The evaluation included functional outcomes (BCVA), anatomical outcomes (CST), injection frequency, and safety. Both groups (aflibercept *n* = 29; faricimab *n* = 25) showed significant and comparable improvements in BCVA and CST at 6 months compared to baseline. In particular, the faricimab-treated group required significantly fewer injections than the aflibercept group (mean ~ 1.96 vs. ~2.45 injections; *p* = 0.024), suggesting a potential reduction in treatment burden, while no serious injection-related adverse events were reported in either arm [37]. This reduction in the number of injections with faricimab, while maintaining equivalent functional and anatomical efficacy, seems to be consistent with the hypothesis that dual VEGF-A/Ang-2 inhibition may confer greater therapeutic durability in BRVO as well, potentially translating into an improved treatment burden profile [37]. For this reason, this study may suggest that faricimab could offer similar efficacy with less frequent administration than aflibercept 2.0 mg in the short term for BRVO, a finding that is potentially relevant for real-world management strategies where treatment burden is a key determinant of patient adherence and satisfaction [37]. However, the lack of randomization and prospective control limits the ability to draw causal conclusions.

Kondo et al. described the protocol of the RVOSTAR study, a prospective, multicenter, single-arm trial designed to evaluate a modified T&E regimen with faricimab for macular edema secondary to CRVO or hemiretinal vein occlusion (HRVO) in naïve Japanese patients [38]. The design aims to study both long-term efficacy (up to 72 weeks) in terms of change in BCVA and CST, as well as factors influencing treatment intervals and the safety of the modified T&E regimen. The RVOSTAR protocol represents an important step towards personalized optimization of faricimab therapy in CRVO, proposing a T&E regimen that includes an induction phase with monthly dosing until edema resolution, a treatment-free observation period aimed at early identification of recurrence via CST, and a T&E maintenance phase with interval extensions or reductions based on objective anatomical criteria without a predetermined maximum interval between injections [38]. This approach is more flexible and potentially more in line with actual clinical practice, as it can reduce the burden of treatment and improve patient adherence [38]. However, no clinical data are currently available, as patient recruitment is still ongoing.

In the FOCUS study by Agrawal et al., real-world experience with faricimab was based on a very limited subgroup of patients with macular edema secondary to RVO, representing approximately 14% of the total cohort (7 eyes) [39]. In this subgroup, faricimab was associated with functional improvements (BCVA) and a reduction in CST during a mean follow-up of approximately 8 months, with no reports of relevant ocular adverse events [39]. Although these results are consistent with the anti-edema efficacy observed in registration trials of faricimab for RVO [32–34], the contribution of the FOCUS study to the specific literature on RVO remains limited and predominantly descriptive. In fact, the extremely small sample size does not allow for robust statistical analysis or a meaningful distinction between RVO subtypes (BRVO vs. CRVO), nor does it allow for reliable assessment of response duration or potential benefit in terms of therapeutic burden [39]. For these reasons, the RVO data from the FOCUS study can be interpreted as a preliminary signal of the efficacy and safety of faricimab in a real-world setting, but they do not add substantial evidence to what has already been demonstrated in phase III randomized trials [32–34].

Hirakata and colleagues reported a short-term retrospective real-world analysis of the efficacy of faricimab in the treatment of macular edema secondary to RVO, including both BRVO and CRVO, in 30 patients (17 naïve, 13 switch) [40]. The study focused primarily on functional (BCVA) and anatomical (CST) outcomes in the first weeks/months of treatment, reflecting an early phase of drug use in clinical practice. The results showed that faricimab can induce rapid decrease of macular edema and significant improvement in visual function in the short term, with a favorable safety profile and no relevant ocular adverse events. These outcomes are consistent with those observed in the registration trials of faricimab in RVO [32–34], suggesting that the efficacy observed in controlled settings is also reproducible in the real world, at least in the initial phase of treatment. Moreover, this study also provided preliminary real-world evidence that is complementary but not comparable to data from phase III trials [32–34] or dedicated prospective protocols (e.g., RVOSTAR) [36]. In fact, compared to these studies, Hirakata et al. did not provide information on the medium- to long-term sustainability of treatment or on the real potential of faricimab to reduce the number of injections, but they hypothesized that the drug could be effective and well tolerated in the early stages, even in unselected patients [40].

In a retrospective monocenter study, Inokuchi et al. reported the short-term real-world results of intravitreal faricimab use in 23 RVO patients, including both BRVO (17 patients) and CRVO (6 patients), with a specific focus on the analysis of optical coherence tomography (OCT) biomarkers as potential predictors of therapeutic response [41]. In terms of primary outcomes, faricimab resulted in significant functional improvement in BCVA and a significant decrease in CST in the short term, confirming potential high early anatomical efficacy. These results are consistent with those reported in other recent real-world studies [37–40] and with data from registration clinical trials [32–34], suggesting that the initial response observed in controlled settings seems to be reproducible in everyday clinical practice. The distinctive feature of this study was the detailed analysis of OCT biomarkers. In particular, the presence of intraretinal fluid and its rapid resolution after treatment were associated with better anatomical outcomes, while some structural signs indicative of chronic damage (such as alterations in the inner retinal layers) appeared to limit visual recovery, despite the macular edema decrease [41]. These data reinforce the idea, already emerging in the literature, that in RVO the dissociation between anatomical and functional response is frequent and that pre-existing ischemic or structural damage could play a crucial role in determining the final visual recovery [41].

The two retrospective studies carried out by Hafner et al. tried to address a clinically relevant and still poorly standardized issue: the role of faricimab as a switch therapy in RVO patients resistant or suboptimal to previous intravitreal treatments [42, 43]. The first study reported initial real-world results on 19 RVO patients [42], while the second showed an extension with follow-up of up to 9 months, allowing for a preliminary assessment of the durability of the response [43]. Overall, these two studies demonstrated that switching to faricimab in patients with therapy-resistant RVO macular edema was associated with significant and sustained anatomical improvement for up to 9 months, while functional benefit remained limited, likely due to pre-existing ischemic and structural damage [42, 43]. These data may suggest that the primary role of faricimab in this setting is anatomical stabilization of the disease rather than visual recovery and support its use as a switch option in selected cases.

In another retrospective study, Hikichi et al. discussed the short-term real-world results of switching to faricimab in 42 RVO patients (23 BRVO, 17 CRVO, 2 HRVO) previously treated with aflibercept 2.0 mg, in a context of suboptimal or insufficient response [44]. The results showed that switching to faricimab is associated with a significant decrease in macular edema in the short term, confirming an early anatomical response even in eyes already exposed to multiple anti-VEGF treatments. In contrast, functional improvement is limited or insignificant, suggesting a frequent dissociation between anatomical and visual response, a phenomenon well known in chronic RVOs and already observed in other switch studies [42, 43]. This dissociation is mainly attributable to pre-existing structural damage and possible macular ischemia, which may prevent visual acuity improvement despite resolution of exudation. This response pattern is consistent with the hypothesis that the main potential benefit of faricimab in previously treated patients could be greater vascular stability and reduced permeability, rather than late visual recovery. The presence of irreversible ischemic and structural damage, typical of chronic or therapy-resistant cases, probably may limit functional response, even in the presence of evident anatomical improvement [44].

Sun et al. described a short-term real-world experience with switching to faricimab in 52 Chinese patients (18 BRVO, 34 CRVO) affected by macular edema secondary to RVO refractory to previous intravitreal therapies, reflecting a clinically complex population characterized by long disease duration and suboptimal response to previous treatments [45]. This study stated that switching to faricimab is associated with a significant CST decrease in the short term, confirming an early anatomical response even in eyes previously considered non-responders or partial responders [45]. On the other hand, functional improvement is modest or not statistically significant, highlighting once again the frequent dissociation between anatomical and visual outcomes in chronic and refractory RVO [42–44].

Hanson and colleagues described a case report on the use of faricimab in an 83-year-old male patient with a diagnosis of BRVO and with refractory multifactorial cystoid macular edema (CME), previously unresponsive or partially responsive to multiple standard therapies, including traditional intravitreal anti-VEGF, intravitreal corticosteroids, and topical anti-inflammation drops [46]. After 3 faricimab injections, CME resolved with a CST change from 748 μm to 339 μm and visual acuity improved from 20/50 to 20/40, showing the efficacy of faricimab in the treatment of CME resistant to other therapies and the importance of continued attempts at using new agents for chronic, recurrent CME [46].

Hikichi et al. reported a comparative study evaluating the 6-month outcomes of aflibercept 2.0 mg and faricimab in treatment-naïve patients with macular edema secondary to BRVO [47]. At 6 months, both treatment groups showed a significant improvement in BCVA together with a marked reduction in CST. Functional and anatomical outcomes were comparable between aflibercept 2.0 mg and faricimab, while the number of injections required during follow-up appeared to be slightly lower in the faricimab group. These findings seem to suggest that faricimab may provide similar short-term efficacy to aflibercept 2.0 mg in treatment-naïve BRVO patients, with a potential reduction in treatment burden.

Wu et al. reported the early real-world experience with faricimab in a large multicenter Latin American cohort (FARI-LATAM study), including patients treated for different retinal diseases, among which RVO represented a relevant subgroup (50 CRVO and 20 BRVO) [48]. Short-term follow-up showed an improvement in BCVA associated with a significant reduction in CST in patients treated with faricimab. Although the study included a heterogeneous population and did not focus exclusively on RVO, the results provided additional real-world evidence supporting the potential anatomical efficacy of faricimab and its clinical applicability in routine practice, including patients with RVO.

Kilani et al. conducted a prospective real-world study evaluating the short-term outcomes of switching to faricimab in patients with anti-VEGF-refractory RVO [49]. The study included previously treated RVO eyes with persistent or recurrent macular edema despite prior anti-VEGF therapy. During the short-term follow-up, a significant reduction in CST was observed in most patients, indicating a good anatomical response after switching therapy. In contrast, functional outcomes were more limited, with BCVA remaining stable in most cases or showing only modest improvement [49].

Finally, Çakır et al. reported the 6-month real-world outcomes of faricimab in a large multicenter cohort in Turkey (FARTURK study) [50]. The study included patients with RVO treated with faricimab in routine clinical practice, including both treatment-naïve and previously treated eyes. At 6 months, patients showed an improvement in BCVA associated with a significant reduction in CST. The anatomical response appeared to be consistent across both naïve and previously treated eyes, while visual improvement was more variable, particularly in chronic cases. Overall, the study provided further real-world evidence confirming the short-term efficacy of faricimab in RVO patients treated in routine clinical practice.

All the discussed studies are briefly summarized in Table 1.

**Table 1:** Summary of the main real-world clinical studies evaluating faricimab in retinal vein occlusion

Study	Design	Population (RVO type)	Setting	Follow-up	Concomitant therapy	Outcomes	Key Results
Hikichi et al. 2025 [37]	Retrospective	54 eyes (BRVO) 29 treated with aflibercept 2.0 mg 25 treated with faricimab 6.0 mg	Naïve	1, 3 and 6 months	None	BCVA, CST, injections	Similar BCVA/CST gains vs. aflibercept 2.0 mg; fewer injections with faricimab
Kondo et al. 2025 [38]	Prospective, single-arm (protocol)	patients are still being recruited (CRVO ± HRVO)	Naïve	Until 72 weeks (study design only)	None	BCVA, CST, durability	Study design only; long-term T&E durability under investigation
Agrawal et al. 2025 [39]	Retrospective	7 RVO eyes (not specified)	Mixed (Naïve + switch)	Minimum follow-up period of 24 weeks	None	BCVA, CST	Both anatomical and functional improvement, but very small sample sample
Hirakata et al. 2025 [40]	Retrospective	30 eyes (20 BRVO 8 CRVO 2 HRVO)	Mixed (Naïve + switch)	Mean follow-up period of 3.7 ± 2.7 and 4.9 ± 2.9 months in the naïve and switch patients, respectively.	Focal laser photocoagulation of the non-perfusion area in 2 BRVO patients in the naïve group and 3 patients (1 BRVO, 1 CRVO, and 1 hemi-CRVO) in the switch group, prior to the initiation of faricimab treatment	BCVA, CST	CST improved in all patients; BCVA gain limited to naïve patients
Inokuchi et al. 2025 [41]	Retrospective	23 eyes (17 BRVO 6 CRVO)	Mixed (Naïve + switch)	At least 3 months	None	BCVA, CST, OCT biomarkers	Early anatomical response; functional outcomes variable
Hafner et al. 2025 [42]	Retrospective	19 eyes (not specified)	Switch	1, 2 and 3 months	Prior to the switch, retinal laser photocoagulation was applied in 10 patients who had responded particularly poorly to previous therapies	CST, BCVA	Marked anatomical improvement; minimal visual gain
Hafner et al. 2025 [43]	Retrospective	19 eyes (not specified)	Switch	At least 9 months	Prior to the switch, retinal laser photocoagulation was applied in 10 patients who had responded particularly poorly to previous therapies	CST durability, BCVA	Anatomical benefit sustained up to 9 months; BCVA stable
Hikichi et al. 2025 [44]	Retrospective	42 eyes (23 BRVO 17 CRVO 2 HRVO)	Switch	Longer than six months	None	CST, BCVA	Short-term CST decrease; limited visual improvement
Sun et al. 2025 [45]	Retrospective	52 eyes (18 BRVO 34 CRVO)	Switch	Mean follow-up duration of 25.65 ± 11.87 weeks	None	CST, BCVA	Significant anatomical response; modest functional benefit
Hanson et al. 2025 [46]	Case report	83-year-old male patient with BRVO	Switch	None	Grid laser therapy was performed	CST, BCVA	Efficacy of faricimab in treatment of cystoid macular edema resistant to other therapies
Hikichi et al. 2026 [47]	Retrospective	54 eyes (BRVO) 29 treated with aflibercept 2.0 mg 25 treated with faricimab 6.0 mg	Naïve	1, 3 and 6 months	None	BCVA, CST, injections	Similar BCVA and CST improvement vs. aflibercept 2.0 mg; slightly lower number of injections with faricimab
Wu et al. 2026 [48]	Retrospective	70 eyes (29 naïve CRVO, 21 switch CRVO, 20 switch BRVO)	Mixed (Naïve + switch)	A mean follow-up of 26.2 weeks for naïve CRVO eyes; a mean follow-up of 21.8 weeks for previously treated CRVO eyes; a mean follow-up of 28.1 weeks for previously-treated BRVO eyes	None	CST, BCVA	Early real-world results showed BCVA improvement and CST reduction; effectiveness confirmed in routine clinical practice including RVO patients
Kilani et al. 2026 [49]	Prospective	27 eyes (12 CRVO and 15 BRVO)	Switch	A mean follow-up of 11.7 weeks	None	BCVA, CST	Significant anatomical improvement after switching to faricimab; limited or modest visual gain during short-term follow-up
Çakır et al. 2026 [50]	Retrospective	21 RVO eyes	Mixed (Naïve + switch)	1 to 6 months	None	BCVA, CST	Improvement in BCVA associated with significant CST reduction at 6 months; anatomical response more consistent than functional improvement in chronic cases

*RVO* retinal vein occlusion, *BRVO* branch retinal vein occlusion, *CRVO* central retinal vein occlusion, *HRVO* hemiretinal vein occlusion, *BCVA* best corrected visual acuity, *CST* central subfoveal thickness, *T&E* treat and extend, *OCT* optical coherence tomography

## Discussion

This systematic review summarizes the current clinical evidence regarding the use of faricimab for the treatment of macular edema secondary to RVO, integrating data from pivotal phase III trials and increasing evidence of real-world studies. Overall, the available literature suggests that faricimab may represent an effective therapeutic option for RVO-related macular edema, with substantial anatomical efficacy and visual outcomes comparable to established anti-VEGF agents, while potentially offering improved treatment durability in selected clinical settings.

The strongest evidence supporting the use of faricimab in RVO derives from the BALATON and COMINO phase III trials [32–34], which demonstrated non-inferior visual outcomes compared to aflibercept 2.0 mg during the fixed monthly dosing phase [32, 33] and sustained functional and anatomical benefits up to 72 weeks within a T&E regimen [34]. These trials contribute important evidence to the evolving management of RVO, as they provide the first randomized evidence that dual VEGF-A and Ang-2 inhibition can maintain long-term disease control with extended treatment intervals. However, although a proportion of patients in the extension phase of BALATON and COMINO were able to reach dosing intervals of up to 12 weeks [34], the absence of a comparative control arm in this phase does not allow a direct evaluation of durability versus other anti-VEGF agents within a T&E regimen. Therefore, while these findings suggest the possibility of extended treatment intervals in a subset of patients, superiority in durability compared with other anti-VEGF therapies remains to be demonstrated.

Conversely, the visual outcomes observed in the phase III BALATON and COMINO trials appear broadly comparable with those historically reported in the pivotal anti-VEGF trials for RVO. In the BRAVO study (BRVO) [51], ranibizumab treatment resulted in a mean visual gain of approximately + 18.3 ETDRS letters at 6 months, while in the CRUISE trial (CRVO) [52], the mean improvement was approximately + 14.9 letters at the same time point. Similarly, the phase III aflibercept trials COPERNICUS [53] and GALILEO [54] reported mean visual gains of approximately + 17 to + 18 letters at 24 weeks, confirming the high efficacy of anti-VEGF therapy in treatment-naïve CRVO patients. In this context, the 24-week results of the BALATON [33] and COMINO [33] trials showed that faricimab achieved visual gains comparable to those obtained with aflibercept and ranibizumab, with non-inferiority demonstrated for the primary endpoint of BCVA improvement. Taken together, these findings suggest that the short-term functional efficacy of faricimab appears broadly consistent with that historically reported for other anti-VEGF agents in RVO (Table 2), although longer follow-up is required to clarify whether differences in durability or treatment burden may emerge over time.

**Table 2:** Main randomized clinical trials of anti-VEGF therapy for macular edema secondary to retinal vein occlusion

Trial	Drug	RVO type	Design	Sample size	Primary endpoint	BCVA gain at 6 months (approx.)	Key findings
BALATON [33]	Faricimab vs. aflibercept	BRVO	Phase 3, randomized, double-masked, active-controlled	553 patients	Change in BCVA at week 24	~+16.9 letters (faricimab)	Faricimab demonstrated non-inferiority to aflibercept with similar functional and anatomical outcomes
COMINO [33]	Faricimab vs. aflibercept	CRVO/HRVO	Phase 3, randomized, double-masked, active-controlled	729 patients	Change in BCVA at week 24	~+16.9 letters (faricimab)	Similar visual outcomes between faricimab and aflibercept; durability evaluated in the extension phase
BRAVO [51]	Ranibizumab vs. sham	BRVO	Phase 3, randomized, double-masked	397 patients	Change in BCVA at month 6	+ 18.3 letters (ranibizumab 0.5 mg)	Significant visual improvement compared with sham; pivotal trial for BRVO
CRUISE [52]	Ranibizumab vs. sham	CRVO	Phase 3, randomized, double-masked	392 patients	Change in BCVA at month 6	+ 14.9 letters (ranibizumab 0.5 mg)	Significant improvement in BCVA compared with sham; pivotal trial for CRVO
COPERNICUS [53]	Aflibercept vs. sham	CRVO	Phase 3, randomized, double-masked	189 patients	Change in BCVA at 24 weeks	~+17.3 letters	Marked visual improvement and high proportion of ≥ 15-letter gain
GALILEO [54]	Aflibercept vs. sham	CRVO	Phase 3, randomized, double-masked	177 patients	Change in BCVA at 24 weeks	~+18 letters	Confirmed efficacy of aflibercept in treatment-naïve CRVO patients

*RVO* retinal vein occlusion, *BRVO* branch retinal vein occlusion, *CRVO* central retinal vein occlusion, *HRVO* hemiretinal vein occlusion, *BCVA* best corrected visual acuity

Beyond randomized clinical trials, real-world evidence plays a crucial role in defining the clinical positioning of faricimab in everyday practice [37–50].

Across the available real-world studies evaluating faricimab for macular edema secondary to RVO, a relatively consistent trend was observed in both functional and anatomical outcomes. BCVA improvement generally ranged from approximately 0.40 logMAR to 0.10 logMAR, with greater gains consistently reported in treatment-naïve cohorts compared with previously treated or refractory eyes. Anatomical response appeared more uniform across studies, with a reduction in CST typically ranging from − 40 to − 416 μm.

The duration of follow-up in the available studies was relatively short, ranging from 1 to 12 months, and most studies reported a limited number of injections during this period, generally between 2 and 7 intravitreal injections. Overall, while functional outcomes appeared more variable—particularly in chronic or anti-VEGF–refractory eyes—anatomical improvement was consistently reported across studies, supporting the potential short-term effectiveness of faricimab in real-world clinical practice.

A relevant proportion of the available real-world evidence specifically focused on patients switched to faricimab because of persistent or recurrent macular edema despite previous anti-VEGF therapy [40–46, 49]. Across these studies, switch populations generally represented chronic and heavily pretreated eyes, frequently characterized by incomplete anatomical response despite multiple prior injections. Overall, approximately 70% of the included patients in these real-world cohorts were previously treated or considered therapy-resistant, depending on study design and inclusion criteria.

Despite the heterogeneity in the definition of refractoriness and the small sample sizes, most studies consistently reported significant anatomical improvement after switching to faricimab, particularly in terms of CST reduction. Across the real-world switch studies reporting anatomical response rates, favorable anatomical outcomes after switching to faricimab were observed in approximately 75,9% of treated eyes (ranging from 44,4% to 100%), although comparisons remain limited by heterogeneous definitions of refractoriness and outcome measures.

However, functional improvement remained limited in many chronic cases, and a subset of eyes continued to show persistent or recurrent fluid despite treatment (approximately 24.1%). Although the exact proportion of anatomical non-responders was inconsistently reported across studies, available evidence seems to suggest that complete anatomical resolution was not achieved in all switched eyes, highlighting the complexity of chronic refractory RVO and the need for standardized response criteria in future studies.

An additional contribution of the reviewed literature is the emerging role of OCT biomarkers in predicting treatment response [41]. Preliminary evidence indicates that eyes with predominantly intraretinal fluid and less structural disruption may derive greater functional benefit, whereas chronic changes in the inner retinal layers limit visual recovery despite edema resolution [41]. These observations reinforce the concept that anatomical success does not necessarily translate into functional gain, thus highlighting the need for individualized treatment strategies based on baseline retinal characteristics [41].

The safety profile of faricimab in RVO appears generally consistent with that previously reported for other intravitreal anti-VEGF agents. In the phase III BALATON and COMINO trials, the incidence of ocular and systemic adverse events was comparable between faricimab and aflibercept, with no new safety signals identified [33]. Real-world studies included in this review also reported a favorable short-term safety profile, although the follow-up period was relatively limited. Post-marketing reports have described rare cases of intraocular inflammation and other adverse events [55–57]; however, the currently available evidence remains limited and does not allow firm conclusions regarding long-term safety. Larger studies with longer follow-up are therefore needed to better define the safety profile of faricimab in RVO patients.

In addition, treatment burden represents one of the major challenges in the management of RVO, as patients often require frequent intravitreal injections and long-term follow-up. In this context, the possibility of extending treatment intervals with faricimab may have important implications not only from a clinical perspective but also in terms of healthcare costs and patient burden, as already demonstrated for age-related macular degeneration and diabetic macular edema [58–62]. However, the currently available data do not yet allow a reliable cost-effectiveness analysis for RVO, and the potential economic advantages associated with extended dosing intervals remain to be confirmed in long-term comparative studies.

The strengths of this review include the comprehensive inclusion of both randomized trials and real-world studies, allowing for a broad and clinically meaningful overview of faricimab use in RVO. By integrating data from treatment-naïve and switch populations, as well as short- and medium-term follow-up, this review provides a realistic representation of current clinical practice. Furthermore, the focus on treatment durability and real-world efficacy addresses clinically relevant questions that extend beyond efficacy alone. In addition, this review integrates evidence from phase III trials and the most recent real-world studies, providing a comprehensive overview of current clinical data on faricimab in RVO.

Nevertheless, several limitations must be acknowledged. First, most real-world studies included are retrospective, monocentric, and characterized by small sample sizes, limiting statistical power and generalizability. Second, heterogeneity in study design, follow-up duration, retreatment criteria, and outcome measures precludes formal quantitative comparison or meta-analysis. Third, many studies did not stratify outcomes by RVO subtype or ischemic status, which are known to influence prognosis and treatment response. Therefore, the current level of evidence should be considered low, and the risk of bias remains high. In addition, the anatomical-functional dissociation observed in several switch studies is not specific to faricimab but has already been widely described in chronic RVO treated with other anti-VEGF agents [63]. For this reason, the currently available real-world data should be interpreted primarily as exploratory rather than confirmatory. Finally, a further limitation of this review concerns the search strategy; the literature search was performed using only two major biomedical databases (PubMed and Scopus), and only articles published in English were included because of language restrictions. Although these databases cover the vast majority of the ophthalmology literature, the possibility that additional relevant studies indexed elsewhere or published in other languages were not identified cannot be completely excluded.

## Clinical implications and future perspectives

Faricimab represents a recently introduced therapeutic option whose role in the RVO treatment algorithm still needs to be clearly defined, particularly considering its dual inhibition of VEGF-A and Ang-2 and its potential for extended dosing intervals. In treatment-naïve patients, especially in earlier stages of disease, faricimab may offer effective anatomical and functional outcomes while allowing for individualized T&E strategies that could reduce treatment burden and improve long-term adherence. In routine clinical practice, this aspect is particularly relevant given the chronic nature of RVO and the well-recognized challenges related to frequent intravitreal injections.

In previously treated or therapy-resistant patients, current real-world data seem to indicate that switching to faricimab is primarily associated with anatomical stabilization rather than substantial visual recovery. This observation highlights the importance of careful patient selection and realistic expectation setting, especially in eyes with longstanding disease, macular ischemia, or irreversible structural damage. In this context, faricimab may be best positioned as a strategy to improve vascular stability and control exudation, rather than to recover visual function in advanced stages of RVO.

However, although faricimab targets both VEGF-A and Ang-2, the currently available clinical trials in RVO do not provide biomarker data or subgroup analyses specifically demonstrating an Ang-2–related clinical benefit. In particular, no analyses have been performed comparing ischemic versus non-ischemic RVO or other clinically relevant subgroups that could support a mechanistic advantage related to Ang-2 inhibition. Therefore, the potential clinical relevance of dual VEGF-A/Ang-2 inhibition in RVO remains largely theoretical at the present time.

Based on the currently available evidence, faricimab can be considered an effective therapeutic option for macular edema secondary to RVO, with functional and anatomical outcomes comparable to those reported with other anti-VEGF agents. At present, its role appears particularly relevant in patients requiring frequent injections or in those with an incomplete anatomical response to previous anti-VEGF therapy. However, the available evidence is still limited, and further comparative studies are required before faricimab can be further clearly positioned as a first-line treatment in all RVO patients.

Looking forward, further prospective studies are needed to better define the optimal timing for faricimab initiation, to identify predictive biomarkers of response, and to clarify its comparative effectiveness versus other anti-VEGF agents under T&E regimens. Long-term real-world data will be essential to assess durability, safety, and treatment burden reduction beyond the first year. Additionally, future research should focus on stratifying outcomes according to RVO subtype, ischemic status, and baseline retinal morphology, with the aim of refining personalized treatment algorithms and maximizing clinical benefit for individual patients.

## Conclusion

In conclusion, current evidence suggests that faricimab may be associated with favorable anatomical outcomes and an acceptable safety profile in the treatment of RVO-related macular edema. Its dual mechanism of action may confer advantages in terms of vascular stabilization and treatment durability, particularly in treatment-naïve patients. In therapy-resistant cases, faricimab appears to provide meaningful anatomical benefit, although functional visual recovery remains limited by disease chronicity and irreversible retinal damage.

Future prospective, head-to-head comparative studies and long-term real-world data are strongly warranted to better define the optimal timing, patient selection, and role of faricimab within the therapeutic algorithm for RVO. Furthermore, future studies should also specifically investigate whether Ang-2 inhibition translates into a clinically meaningful advantage in selected RVO subgroups, such as ischemic RVO or patients with persistent inflammatory or vascular instability features, as this aspect remains insufficiently explored in the current literature.

## Data Availability

No datasets were generated or analysed during the current study.
